# Supplementary figures and images for: Dendritic cell entry to lymphatic capillaries is orchestrated by CD44 and the hyaluronan glycocalyx
Source: Life Sci Alliance. 2021 Mar 9;4(5):e202000908. doi: 10.26508/lsa.202000908 (PMC8008951; doi:10.26508/lsa.202000908)

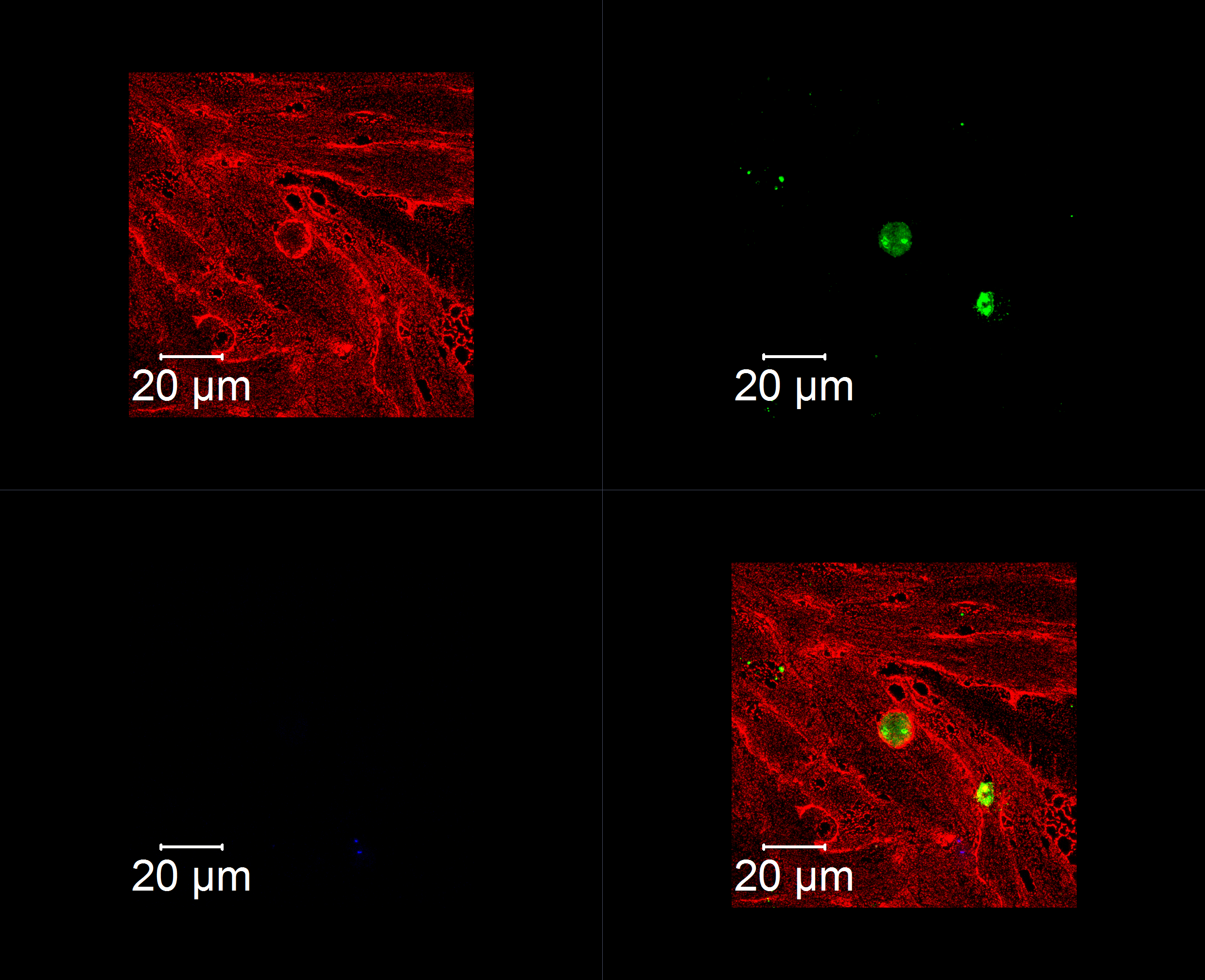

Supplement: Supplementary file 10 [file LSA-2020-00908_SdataF9.3.tif]

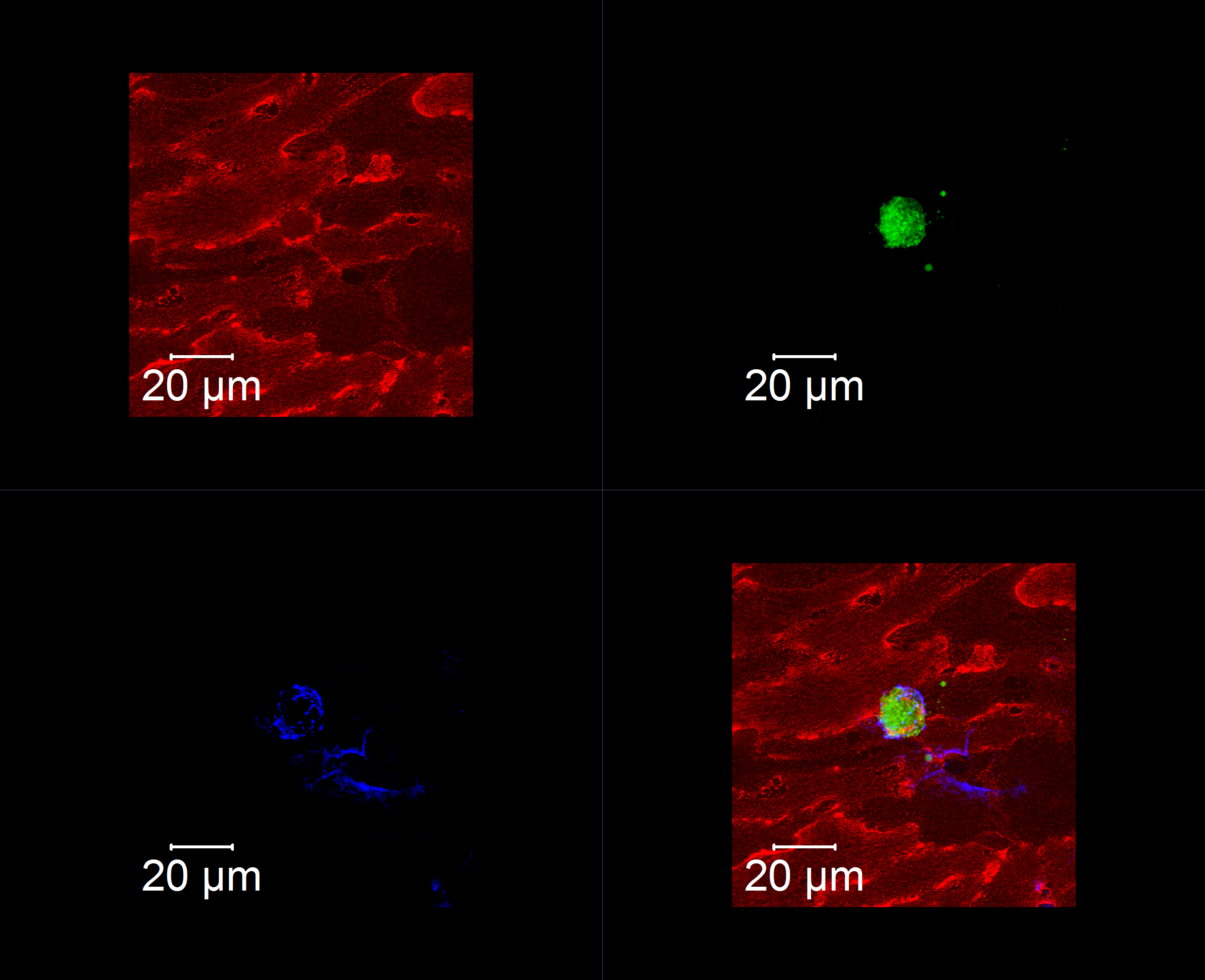

Supplement: Supplementary file 11 [file LSA-2020-00908_SdataF9.4.tif]
